# Non-contiguous finished genome sequence and description of *Brevibacillus massiliensis* sp. nov.

**DOI:** 10.4056/sigs.3466975

**Published:** 2013-04-15

**Authors:** Perrine Hugon, Ajay Kumar Mishra, Jean-Christophe Lagier, Thi Thien Nguyen, Carine Couderc, Didier Raoult, Pierre-Edouard Fournier

**Affiliations:** 1 Aix-Marseille Université, URMITE, Faculté de médecine, France

**Keywords:** *Brevibacillus massiliensis*, genome, culturomics, taxono-genomics

## Abstract

*Brevibacillus massiliensis* strain phR^T^ sp. nov. is the type strain of *B. massiliensis* sp. nov., a new species within the genus *Brevibacillus*. This strain was isolated from the fecal flora of a woman suffering from morbid obesity. *B. massiliensis* is a Gram-positive aerobic rod-shaped bacterium. Here we describe the features of this organism, together with the complete genome sequence and annotation. The 5,051,018 bp long genome (1 chromosome but no plasmid) contains 5,051 protein-coding and 84 RNA genes, and exhibits a G+C content of 53.1%.

## Introduction

*Brevibacillus massiliensis* strain phR^T^ (= CSUR P177 = DSM 25447) is the type strain of *B. massiliensis* sp. nov. This bacterium is a Gram-positive, spore-forming, indole negative, aerobic and motile bacillus that was isolated from the stool of a 26-year-old woman suffering from morbid obesity. The strain was isolated as part of a study aiming at individually cultivating all species within human feces [1]. The current approach to classification of prokaryotes, often referred to as polyphasic taxonomy, relies on a combination of phenotypic and genotypic characteristics [2]. However, as more than 3,000 bacterial genomes have been sequenced to date [3] and the cost of genomic sequencing is decreasing, we recently proposed to integrate genomic information in the description of new bacterial species [4-15].

The genus *Brevibacillus* (Shilda *et al*. 1996) was created in 1996 by reclassification of 10 *Bacillus* species, on the basis of 16S rDNA gene sequence analysis [16]. To date, this genus is made of 18 species [17], including *B. agri, B. brevis, B. centrosporus, B. choshinensis, B. parabrevis, B. reuszeri*, *B. formosus, B. borstelensis, B. laterosporus*, and *B. thermoruber* [16], *B. invocatus* [18], *B. limnophilus* [19], *B. levickii* [20], *B. ginsengisoli* [21], *B. panacihumi* in [22], *B. fluminis* in [23], and *B. nitrificans* [24]. Members of the genus *Brevibacillus* are environmental bacteria and were mostly isolated from soil [22,25]. In addition, *B. brevis* and *B. centrosporus* were isolated from indoor dust in schools, day care centers for children and animal sheds [26], and fecal flora of children, respectively [27]. However, several *Brevibacillus* species are also frequently isolated from humans, notably in nosocomial infections, causing breast abscess, pneumonia [18], peritonitis [28] and endopthalmitis [29].

Here we present a summary classification and a set of features for *B. massiliensis* sp. nov. strain phR^T^ (= CSUR P177 = DSM 25447), together with the description of the complete genomic sequencing and annotation. These characteristics support the circumscription of the *B. massiliensis* species.

## Classification and features

A stool sample was collected from a 26-year-old woman living in Marseille (France). She suffered from morbid obesity and had a body mass index of 48.2 (118.8 kg, 1.57 meter). At the time of stool sample collection she was not under medication or on a diet. The patient gave an informed and signed consent. This study and the assent procedure were approved by the Ethics Committee of the Institut Fédératif de Recherche IFR48, Faculty of Medicine, Marseille, France (agreement 11-017). The fecal specimen was preserved at -80°C after collection. Strain phR^T^ (Table 1) was isolated in 2011 by aerobic cultivation on M17 agar medium (Oxoid, Basingstoke, England).

**Table 1:** Classification and general features of *Brevibacillus massiliensis* strain phR^T^ according to the MIGS recommendations [42]

**MIGS ID**	**Property**	**Term**	**Evidence code^a^**
		Domain *Bacteria*	TAS [30]
		Phylum *Firmicutes*	TAS [31-33]
		Class *Bacilli*	TAS [34,35]
	Current classification	Order *Bacillales*	TAS [36,37]
		Family *Paenibacillaceae*	TAS [34,38]
		Genus *Brevibacillus*	TAS [16]
		Species *Brevibacillus massiliensis*	IDA
		Type strain phR^T^	IDA
	Gram stain	positive	IDA
	Cell shape	rod	IDA
	Motility	motile	IDA
	Sporulation	sporulating	IDA
	Temperature range	mesophile	IDA
	Optimum temperature	37°C	IDA
MIGS-6.3	Salinity	growth in BHI medium + 5% NaCl	IDA
MIGS-22	Oxygen requirement	aerobic	IDA
	Carbon source	unknown	
	Energy source	unknown	
MIGS-6	Habitat	human gut	IDA
MIGS-15	Biotic relationship	free living	IDA
MIGS-14	Pathogenicity Biosafety level Isolation	unknown 2 human feces	
MIGS-4	Geographic location	France	IDA
MIGS-5	Sample collection time	January 2011	IDA
MIGS-4.1	Latitude	43.296482	IDA
MIGS-4.2	Longitude	5.36978	IDA
MIGS-4.3	Depth	surface	IDA
MIGS-4.4	Altitude	0 m above sea level	IDA

Evidence codes - IDA: Inferred from Direct Assay; TAS: Traceable Author Statement (i.e., a direct report exists in the literature); NAS: Non-traceable Author Statement (i.e., not directly observed for the living, isolated sample, but based on a generally accepted property for the species, or anecdotal evidence). These evidence codes are from the Gene Ontology project [39]. If the evidence is IDA, then the property was directly observed for a live isolate by one of the authors or an expert mentioned in the acknowledgements.

**Figure 1:**
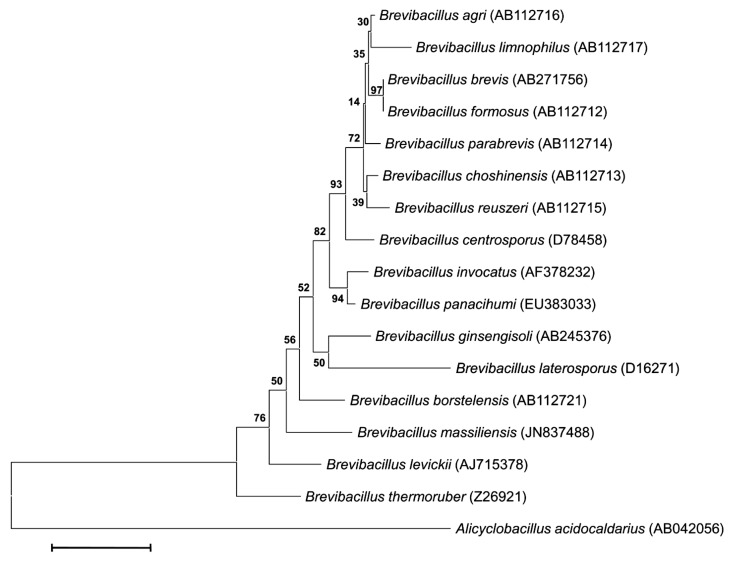
Phylogenetic tree highlighting the position of *Brevibacillus massiliensis* strain phR^T^ relative to other type strains within the *Brevibacillus* genus. GenBank accession numbers are indicated in parentheses. Sequences were aligned using CLUSTALW, and phylogenetic inferences obtained using the maximum-likelihood method within MEGA program. Numbers at the nodes are percentages of bootstrap values obtained by repeating the analysis 500 times to generate a majority consensus tree. *Alicyclobacillus acidocaldarius* was used as outgroup. The scale bar represents a 2% nucleotide sequence divergence.

This strain exhibited a nucleotide sequence similarity with *Brevibacillus* species ranging from 94% with *B. centrosporus* [18] to 96% with *B. reuszeri*, *B. parabrevis*, *B. invocatus*, *B. brevis*, *B. borstelensis* [18], *B. panacihumi* [22], *B. levickii* [20], (Figure 1). This latter value was lower than the 98.7% 16S rRNA gene sequence threshold recommended by Stackebrandt and Ebers to delineate a new species without carrying out DNA-DNA hybridization [40].

**Figure 2:**
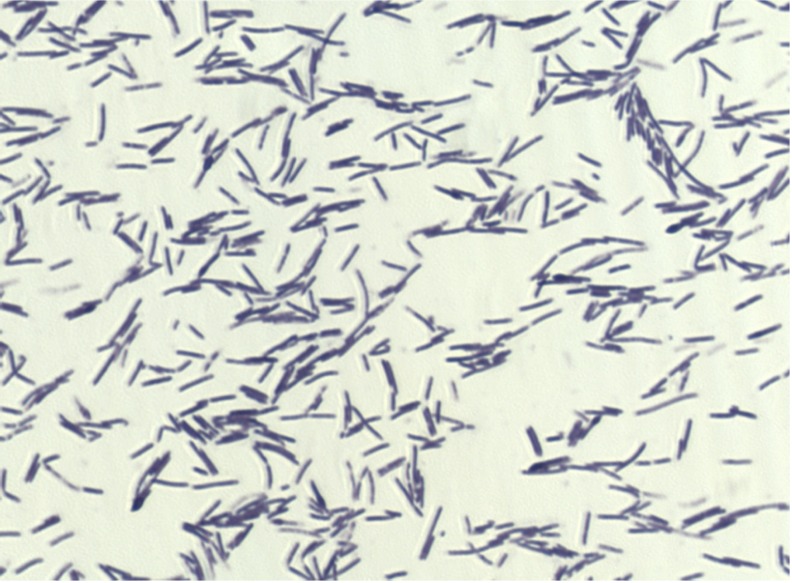
Gram staining of *B. massiliensis* strain phR^T^

**Figure 3:**
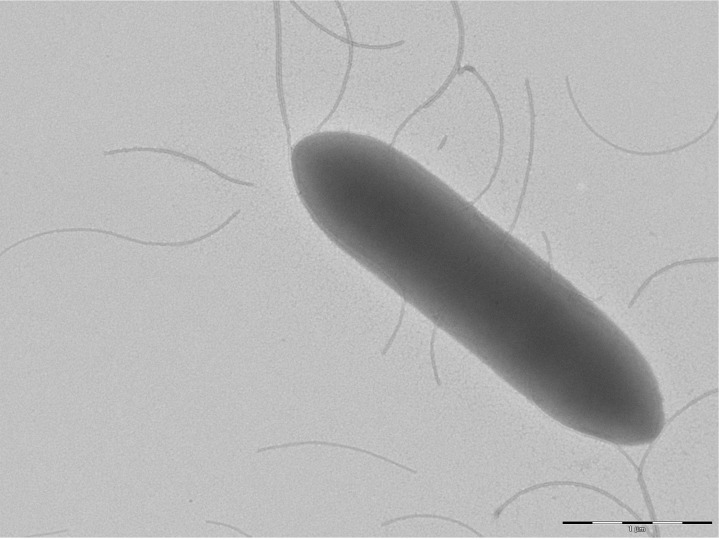
Transmission electron microscopy of *B. massiliensis* strain phR^T^, using a Morgani 268D (Philips) at an operating voltage of 60kV. The scale bar represents 1 µm.

Different growth temperatures (25, 30, 37, 45°C [Table 2]) were tested; no growth occurred at 25°C, growth occurred between 30 and 45°C, and optimal growth was observed at 37°C. Grey colonies were 0.8 mm to 1 mm in diameter on blood-enriched Columbia agar and Brain Heart Infusion (BHI) agar. Growth of the strain was tested under anaerobic and microaerophilic conditions using GENbag anaer and GENbag microaer systems, respectively (BioMerieux), and in the presence of air, with or without 5% CO_2_. Growth was obtained aerobically. A weak growth was observed with 5% CO_2_, but no growth occurred in microaerophilic and anaerobic conditions. Gram staining showed Gram-positive rods (Figure 2). The motility test was positive. Cell diameters ranged from 0.61 µm to 0.80 µm, with a mean diameter of 0.74 µm, and from 2.60µm to 7.30 µm long, with a mean length of 4.3µm in electron microscopy. Peritrichous flagellae were also observed (Figure 3).

**Table 2:** Differential characteristics of *B. massiliensis* sp. nov strain ph1^T^, *B. agri* strain NRRL NRS-1219, *B. laterosporus* strain JCM 2496 and *B. brevis* NBRC 15304^T^.

**Properties**	*B.massiliensis*	*B.agri*	*B.laterosporus*	*B.brevis*
Cell diameter (µm)	0.74	0.75	na	0.50
Oxygen requirement	aerobic	aerobic	aerobic, facultative anaerobic	aerobic
Gram stain	+	+	var	+
Salt requirement	na	na	na	-
Motility	+	+	+	+
Endospore formation	na	+	+	+
**Production of**				
Alkaline phosphatase	+	na	na	-
Acid phosphatase	+	na	na	-
Catalase	+	+	+	+
Oxidase	+	-	na	+
Nitrate reductase	-	-	na	+
Urease	-	-	na	-
α-galactosidase	-	na	na	-
β- galactosidase	-	na	na	-
β-glucuronidase	-	na	na	-
α -glucosidase	-	na	na	-
N-acetyl- β -glucosamidase	-	na	na	-
Indole	-	-	-	-
Esterase	w	na	na	+
Esterase lipase	w	na	na	+
Naphthyl-AS-BI-phosphohydrolase	+	na	na	+
Arginine arylamidase	-	na	na	na
Arginine dihydrolase	-	na	na	-
Glutamyl glutamic acid arylamidase	-	na	na	na
Phenylalanine arylamidase	-	na	na	na
Leucine arylamidase	w	na	na	-
Cystine arylamidase	+	na	na	-
Valine arylamidase	w	na	na	-
Glycine arylamidase	-	na	na	na
Histidine arylamidase	-	na	na	na
Serine arylamidase	-	na	na	na
**Utilization of**				
D-mannose	-	na	na	-
**Habitat**	human gut	environment	environment	environment

Strain phR^T^ exhibited catalase and oxidase activities. Using an API ZYM strip (BioMerieux, Marcy l’Etoile), positive reactions were obtained for alkaline phosphatase, cystine arylamidase, acid phosphatase and naphtol-AS-BI-phosphohydrolase. Weak reactions were obtained for esterase, esterase lipase, leucine arylamidase, valine arylamidase, and α-chymotrypsin. Using an API Coryne strip (BioMerieux), positive reactions were obtained for pyrazinamidase and alkaline phosphatase. No sugar fermentation was observed using API 50CH (Biomerieux).

*B. massiliensis* is susceptible to penicillin G, amoxicillin, amoxicillin + clavulanic acid, ceftriaxon, imipenem, erythromycin, doxycyclin, rifampicine, vancomycin, ciprofloxacin, gentamicin, nitrofurantoin and resistant to metronidazole and trimetoprim + sulfamethoxazole. By comparison with *B. borstelensis*, its phylogenetically-closest neighbor, *B. massiliensis* differed in fumarate, phenylacetate and glutamate activities [18]. By comparison with *B. brevis*, *B.massiliensis* differed in alkaline and acid phosphatase production, nitrate reductase, esterase, esterase lipase, leucine arylamidase, cystine arylamidase and valine arylamidase production. By comparison with *B. agri*, *B. massiliensis* differed in oxidase production.

**Figure 4:**
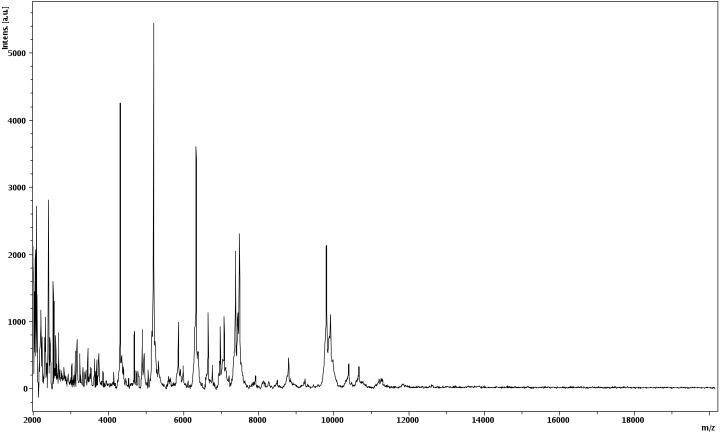
Reference mass spectrum from *B. massiliensis* strain phR^T^. Spectra from 12 individual colonies were compared and a reference spectrum was generated.

**Figure 5:**
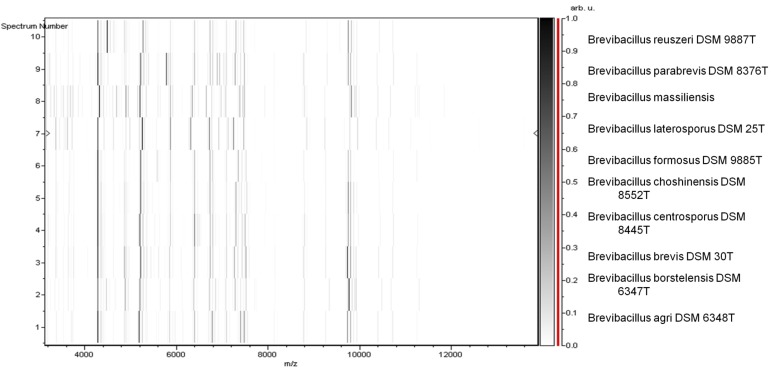
Gel view comparing *Brevibacillus massiliensis* phR^T^ spectra with other members into *Brevibacillus* genera (*Brevibacillus reuszeri*, *Brevibacillus parabrevis*, *Brevibacillus laterosporus*, *Brevibacillus formosus*, *Brevibacillus choshinensis*, *Brevibacillus centrosporus*, *Brevibacillus brevis*, *Brevibacillus borstelensis*, *Brevibacillus agri*). The Gel View displays the raw spectra of all loaded spectrum files arranged in a pseudo-gel like look. The x-axis records the m/z value. The left y-axis displays the running spectrum number originating from subsequent spectra loading. The peak intensity is expressed by a Gray scale scheme code. The color bar and the right y-axis indicates the relation between the color a peak is displayed with and the peak intensity in arbitrary units.

Matrix-assisted laser-desorption/ionization time-of-flight (MALDI-TOF) MS protein analysis was carried out as previously described [41]. Briefly, a pipette tip was used to pick an isolated bacterial colony from a culture agar plate and spread it as a thin film on a MTP 384 MALDI-TOF target plate (Bruker Daltonics, Germany). Twelve distinct deposits were done for strain phR^T^ from twelve isolated colonies. Each smear was overlaid with 2µL of matrix solution (saturated solution of alpha-cyano-4-hydroxycinnamic acid) in 50% acetonitrile, 2.5% tri-fluoracetic acid, and allowed to dry for five minutes. Measurements were performed with a Microflex spectrometer (Bruker). Spectra were recorded in the positive linear mode for the mass range of 2,000 to 20,000 Da (parameter settings: ion source 1 (ISI), 20kV; IS2, 18.5 kV; lens, 7 kV). A spectrum was obtained after 675 shots at a variable laser power. The time of acquisition was between 30 seconds and 1 minute per spot. The twelve phR^T^ spectra were imported into the MALDI BioTyper software (version 2.0, Bruker) and analyzed by standard pattern matching (with default parameter settings) against the main spectra of 3,769 bacteria, including spectra from nine validly published *Brevibacillus* species that were used as reference data in the BioTyper database (updated March 15^th^, 2012). The method of identification includes the m/z from 3,000 to 15,000 Da. For every spectrum, 100 peaks at most were taken into account and compared with the spectra in the database. A score enabled the presumptive identification and discrimination of the tested species from those in a database: a score > 2 with a validated species enabled the identification at the species level; a score > 1.7 but < 2 enabled the identification at the genus level; and a score < 1.7 did not enable any identification. For strain phR^T^, no significance score was obtained, thus suggesting that our isolate was not a member of a known species. We incremented our database with the spectrum from strain phR^T^ (Figure 4). Finally, the gel view allows us to highlight the spectra differences with other of *Brevibacillus* genera members (Figure 5).

## Genome sequencing information

### Genome project history

The organism was selected for sequencing on the basis of its phylogenetic position and 16S rRNA similarity to other members of the *Brevibacillus* genus, and is part of a study of the human digestive flora aiming at isolating all bacterial species within human feces. It was the fifth genome of a *Brevibacillus* species and the first genome of *Brevibacillus massiliensis* sp. nov. The Genbank accession number is CAGW00000000 and consists of 132 contigs. Table 3 shows the project information and its association with MIGS version 2.0 compliance [42].

**Table 3:** Project information

**MIGS ID**	**Property**	**Term**
MIGS-31	Finishing quality	High-quality draft
MIGS-28	Libraries used	One 454 paired end 3-kb library
MIGS-29	Sequencing platforms	454 GS FLX Titanium
MIGS-31.2	Fold coverage	54.2×
MIGS-30	Assemblers	Newbler version 2.5.3
MIGS-32	Gene calling method	Prodigal
	INSDC ID	PRJEA82077
	Genbank ID	CAGW00000000
	Genbank Date of Release	May 30, 2012
	Project relevance	Study of the human gut microbiome

### Growth conditions and DNA isolation

*B. massiliensis* sp. nov. strain phR^T^, (= CSUR P177 = DSM 25447), was grown aerobically on M17 agar medium at 37°C. Five petri dishes were spread and resuspended in 3×100µl of G2 buffer (EZ1 DNA Tissue kit, Qiagen). A first mechanical lysis was performed using glass powder on a Fastprep-24 device (Sample Preparation system, MP Biomedicals, USA) during 2×20 seconds. DNA was then treated with 2.5 µg/µL (30 minutes at 37°C) and extracted using a BioRobot EZ 1 Advanced XL (Qiagen). The DNA was then concentrated and purified on a Qiamp kit (Qiagen). The yield and the concentration was measured by the Quant-it Picogreen kit (Invitrogen) on the Genios_Tecan fluorometer at 36.8 ng/µl.

### Genome sequencing and assembly

A 3kb paired-end sequencing strategy (Roche, Meylan, France) was used. Five µg of DNA was mechanically fragmented on the Hydroshear device (Digilab, Holliston, MA,USA) with an enrichment size at 3-4kb. The DNA fragmentation was visualized through an Agilent 2100 BioAnalyzer on a DNA labchip 7500 with an optimal size of 3.2 kb. The library was constructed according to the 454 GS FLX Titanium paired end protocol. Circularization and nebulization were performed and generated a pattern with an optimal at 555 bp. After PCR amplification through 17 cycles followed by double size selection, the single stranded paired-end library was then quantified on the Quant-it Ribogreen kit (Invitrogen) on the Genios_Tecan fluorometer at 21 pg/µL. The library concentration equivalence was calculated as 6.94e+07 molecules/µL. The library was stored at -20°C until further use.

The 3kb paired-end library was amplified in 9 emPCR reactions at 1cpb, and in 2 emPCRs at 0.5 cpb with the GS Titanium SV emPCR Kit (Lib-L) v2 (Roche).The yield of the 2 types of paired-end emPCR reactions was 7.8% and 11.2%, respectively, in the quality range of 5 to 20% expected from the Roche procedure. Both libraries were loaded onto GS Titanium PicoTiterPlates (PTP Kit 70×75, Roche) and pyrosequenced with the GS Titanium Sequencing Kit XLR70 and the GS FLX Titanium sequencer (Roche).The run was performed overnight and then analyzed on the cluster through the gsRunBrowser and Newbler assembler (Roche). A total of 969,014 passed filter wells were obtained and generated 274 Mb with a length average of 286 bp. The passed filter sequences were assembled using Newbler with 90% identity and 40bp as overlap. The final assembly identified 31 scaffolds and 129 contigs (>1,500 bp) and generated a genome size of 5.05Mb, which corresponds to a coverage of 54.2× coverage.

### Genome annotation

Open Reading Frames (ORFs) were predicted using Prodigal [43] with default parameters but the predicted ORFs were excluded if they spanned a sequencing gap region. The predicted bacterial protein sequences were searched against the GenBank database [44] and the Clusters of Orthologous Groups (COG) databases using BLASTP. The tRNAScanSE tool [45] was used to find tRNA genes, whereas ribosomal RNAs were found by using RNAmmer [46] and BLASTN against the GenBank database. Lipoprotein signal peptides and numbers of transmembrane helices were predicted using SignalP [47] and TMHMM [48], respectively. ORFans were identified if their BLASTP *E*-value was lower than 1e-03 for alignment length greater than 80 amino acids. If alignment lengths were smaller than 80 amino acids, we used an *E*-value of 1e-05. Such parameter thresholds have already been used in previous works to define ORFans. To estimate the mean level of nucleotide sequence similarity at the genome level between *B. massiliensis* strain phR^T^, *B. laterosporus* strain LMG15441 (GenBank accession number AFRV00000000) and *B. brevis* strain NBRC100599 (GenBank accession number AP008955) and *B. agri* strain BAB-2500, we compared genomes two by two and determined the mean percentage of nucleotide sequence identity among orthologous ORFs using BLASTn. Orthologous genes were detected using the Proteinortho software [49].

## Genome properties

**Figure 6:**
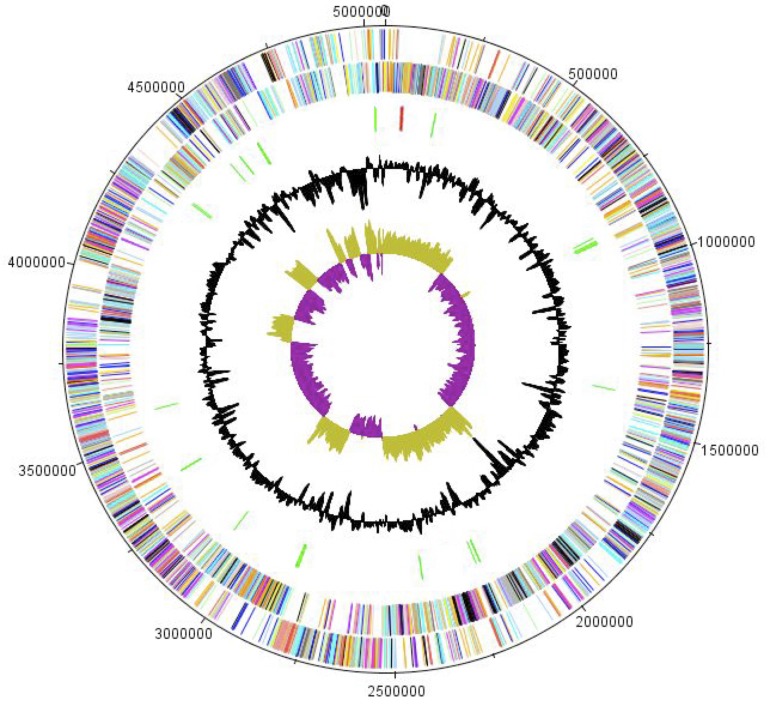
Graphical circular map of the chromosome. From the outside in, the outer two circles shows open reading frames oriented in the forward (colored by COG categories) and reverse (colored by COG categories) direction, respectively. The third circle marks the rRNA gene operon (red) and tRNA genes (green). The fourth circle shows the G+C% content plot. The inner-most circle shows GC skew, purple indicating negative values whereas olive for positive values.

The genome of *B. massiliensis* strain phR^T^ is 5,051,018 bp long (1 chromosome but no plasmid) with a G + C content of 53.1% (Figure 6 and Table 4). Of the 5,135 predicted genes, 5,051 were protein-coding genes, and 84 were RNAs. Three rRNA genes (one 16S rRNA, one 23S rRNA and one 5S rRNA) and 81 predicted tRNA genes were identified in the genome. A total of 3,793 genes (73.86%) were assigned a putative function. Three hundred and seventy-eight genes were identified as ORFans (7.36%). The remaining genes were annotated as hypothetical proteins. The properties and the statistics of the genome are summarized in Table 4. The distribution of genes into COGs functional categories is presented in Table 5.

**Table 4:** Nucleotide content and gene count levels of the genome

**Attribute**	**Value**	**% of total^a^**
Genome size (bp)	5,051,018	
DNA coding region (bp)	4,481,706	88.72
DNA G+C content (bp)	2,682,091	53.10
Number of replicons	1	
Extrachromosomal elements	0	
Total genes	5,135	100
RNA genes	84	1.63
rRNA operons	1	
Protein-coding genes	5,051	98.36
Genes with function prediction	4,198	81.75
Genes assigned to COGs	3,793	73.86
Genes with peptide signals	354	6.89
Genes with transmembrane helices	1,277	24.86
CRISPR repeats	0	

^a^ The total is based on either the size of the genome in base pairs or the total number of protein coding genes in the annotated genome

**Table 5:** Number of genes associated with the 25 general COG functional categories

**Code**	**Value**	**%age**^a^	**Description**
J	165	3.27	Translation
A	0	0	RNA processing and modification
K	405	8.0	Transcription
L	176	3.48	Replication, recombination and repair
B	1	0.02	Chromatin structure and dynamics
D	33	065	Cell cycle control, mitosis and meiosis
Y	0	0	Nuclear structure
V	50	0.99	Defense mechanisms
T	233	4.61	Signal transduction mechanisms
M	181	3.58	Cell wall/membrane biogenesis
N	60	1.19	Cell motility
Z	0	0	Cytoskeleton
W	0	0	Extracellular structures
U	42	0.83	Intracellular trafficking and secretion
O	111	2.20	Posttranslational modification, protein turnover, chaperones
C	251	4.97	Energy production and conversion
G	327	6.47	Carbohydrate transport and metabolism
E	700	13.86	Amino acid transport and metabolism
F	88	1.74	Nucleotide transport and metabolism
H	159	3.15	Coenzyme transport and metabolism
I	171	3.39	Lipid transport and metabolism
P	317	6.28	Inorganic ion transport and metabolism
Q	150	2.97	Secondary metabolites biosynthesis, transport and catabolism
R	578	11.44	General function prediction only
S	319	6.32	Function unknown
-	1,258	24.91	Not in COGs

^a^ The total is based on the total number of protein coding genes in the annotated genome.

## Comparison with other *Brevibacillus* species genomes

Here, we compared the genome of *B. massiliensis* strain phR^T^ with those of *B. laterosporus* strain LMG15441,*B. brevis* strain NBRC100599 and *B. agri* strain BAB-2500. The draft genome of *B. massiliensis* is smaller than those of *B. laterosporus*, *B.agri* and *B. brevis* (5.05, 5.14, 5.39 and 6.29 Mb, respectively). *Brevibacillus massiliensis* has a higher G+C content than *Brevibacillus laterosporus* and *Brevibacillus brevis* (53.10% vs 41.09% and 47.27% respectively) but smaller G+C content than Brevibacillus agri (53.10% vs 53.5%). *B. massiliensis* has a higher gene content than *B. laterosporus* (5,051 and 4,591, respectively) but lower than *B. agri* and *B. brevis* respectively (5.457 and 5.949 respectively). In addition, *B. massiliensis* shared 2,077, 2,500 and 2,453 orthologous genes with *B. laterosporus*, *B. brevis* and *B. agri* respectively. The average nucleotide sequence identity ranged from 67.17 to 78.81% among *Brevibacillus* species, and from 67.34 to 71.14% between *B. massiliensis* and other *Brevibacillus* species, thus confirming its new species status (Table 6).

**Table 6:** The numbers of orthologous protein shared between genomes (above diagonal)^†^

	*B. massiliensis*	*B. laterosporus*	*B. brevis*	*B. agri*
*B. massiliensis*	**5,051**	2,077	2,500	2,453
*B. laterosporus*	67.34	**4,591**	2,403	2,356
*B. brevis*	69.36	68.38	**5,949**	2,779
*B. agri*	71.14	67.17	78.81	**5,457**

^†^average percentage similarity of nucleotides corresponding to orthologous protein shared between genomes (below diagonal) and the numbers of proteins per genome (bold) [49].

## Conclusion

On the basis of phenotypic, phylogenetic and genomic analyses, we formally propose the creation of *Brevibacillus massiliensis* sp. nov. which currently contains strain phR^T^ as its sole member; . This bacterial strain was originally isolated in Marseille, France.

### Description of *Brevibacillus massiliensis* sp. nov.

*Brevibacillus massiliensis* (ma.si.li.en′sis. L. gen. masc. n. *massiliensis*, pertaining to Massilia, the ancient Roman name for Marseille, France, where the type strain was isolated).

Colonies are grey and 0.8 mm to 1 mm in diameter on blood-enriched Columbia agar. Cells are rod-shaped with a mean diameter of 0.74 µm and a mean length of 4.3µm with electron microscopy. Optimal growth is achieved aerobically. Weak growth was observed when cultures were gown under a 5% CO_2_. No growth is observed in microaerophilic or anaerobic conditions. Growth occurs between 30 and 45°C, with optimal growth occurring at 37°C. Cells stain Gram-positive, form endospores and are motile. Cells are positive for catalase, oxidase, alkaline phosphatase, cystine arylamidase, acid phosphatase, naphtol-AS-BI-phosphohydrolase and pyrazinamidase. Asaccharolytic. Cells are susceptible to penicillin G, amoxicillin, amoxicillin + clavulanic acid, ceftriaxone, imipenem, erythromycin, doxycycline, rifampicine, vancomycin, ciprofloxacin, gentamicin, nitrofurantoin and resistant to metronidazole and trimethoprim/sulfamethoxazole. The G+C content of the genome is 53.1%. The 16S rRNA and genome sequences are deposited in Genbank and EMBL under accession numbers JN837488 and CAGW00000000, respectively.

The type strain phR^T^ (= CSUR P177 = DSM 25447) was isolated from the fecal flora of an obese patient in Marseille, France.
